# Prospective application of implementation science theories and frameworks to inform use of PROMs in routine clinical care within an integrated pain network

**DOI:** 10.1007/s11136-020-02600-8

**Published:** 2020-09-02

**Authors:** Sara Ahmed, Diana Zidarov, Owis Eilayyan, Regina Visca

**Affiliations:** 1 Faculty of Medicine, School of Physical & Occupational Therapy, McGill University, Montréal, QC Canada; 2 Center for Outcome Research and Evaluation, Clinical Epidemiology, McGill University Health Center, McGill University, Montréal, QC Canada; 3 Centre de Recherche Interdisciplinaire en réadaptation (CRIR), Constance Lethbridge Rehabilitation Center, Montréal, QC Canada; 4 Institut Universitaire Sur La réadaptation en déficience Physique de Montréal, Centre intégré Universitaire de santé Et de Services Sociaux du Centre-Sud-de-L’Ile-de-Montréal, Montréal, Québec Canada; 5 Faculté de Médecine, Université de Montréal, École de réadaptation, Montréal, Canada; 6 RUISSS McGill Centre of Expertise in Chronic Pain, Montréal, Canada; 7 Department of Family Medicine, Faculty of Medicine, McGill University, Montréal, QC Canada

**Keywords:** Implementation science, Theoretical frameworks, Barriers, Enablers, Patient-reported outcome measures, Chronic pain, Integrated care

## Abstract

**Purpose:**

The objective of this study is to present the implementation science approaches that were used before implementing electronic patient-reported outcome measures (ePROMs) across an integrated chronic pain network that includes primary, rehabilitation, and hospital-based care.

**Methods:**

The Theoretical Domains Framework (TDF) was used to identify potential barriers and enablers to the use of ePROMS by primary care clinicians. In rehabilitation and tertiary care, the Consolidated Framework for Implementation (CFIR) was used to guide the identification of determinants of implementations, through observation of workflow, patient and clinician surveys, and clinician interviews. A mixed-method concurrent design comprising a quantitative and qualitative analysis was used. The results were reviewed by a steering committee to iteratively inform the ePROM implementation plan. The Proctor framework of evaluation was used to guide the development of an evaluation plan for the implementation of ePROMs in the integrated chronic pain network.

**Results:**

Both frameworks provided similar results with respect to healthcare provider knowledge, behaviour, and experience interpreting PROM scores. The TDF and CFIR frameworks differed in identifying organizational-level determinants. The resultant implementation plan was structured around the adoption of PROMs to inform individual treatment planning and quality improvement. The evaluation plan focused on implementation and impact outcomes to evaluate the ePROM intervention.

**Conclusions:**

The TDF and CFIR guided the development of a multi-component knowledge translation and training intervention that will address multiple gaps and barriers to implementation of PROMs across the integrated network. The ePROM intervention will aim to increase clinicians’ knowledge and skills and foster best practices.

## Background

Chronic pain (CP) is a complex condition that affects the individual’s daily activities and quality of life, and social and family environments [1]. Management of CP patients is fragmented, with patients seeking a wide variety of primary and specialty care services [2–5] resulting in poorer outcomes and higher healthcare costs as patients often receive unneeded diagnostic and medical procedures [3, 6]. Pain has the potential to impact a range of domains of a person’s health and quality of life including physical, mental, emotional, and cognitive function. Patient-Reported Outcome Measures (PROMs) enable clinicians to incorporate the patient voice in evaluating the impact of their health condition on their function and health-related quality of life (HRQL), [7–9] and in optimizing treatment planning. There is evidence to support that PROMs are necessary to capture outcomes that are meaningful to individuals with chronic pain and to support a patient-centred care approach [10, 11].

To increase their widespread adoption and to optimize their usefulness to a range of stakeholders, PROMs must be perceived as relevant, meaningful, and actionable to the patients and the clinicians using them and the organizations implementing them. Using implementation research can help identify strategies that address the contextual barriers and facilitators to using PROMs in a specific clinical context. There are over 60 frameworks to guide the implementation of healthcare innovations [12]. Using frameworks to guide the application of PROMs in clinical practice is important as they can facilitate identification of determinants of implementation, guide the selection of implementation strategies, and evaluate if implementation was effective. Frameworks can also guide the selection of domains that influence sustainability of the use of PROMs, and their impact on quality of care and health outcomes, and provide a common language to synthesize results across studies [13].

As part of a 5-year strategic plan, the McGill RUISSS Center of Expertise in Chronic Pain (CECP) developed processes to implement and evaluate the impact of PROMs across an integrated care network. Taking a grass roots approach, the CECP leadership asked chronic pain clinics about their interest in implementing PROMs. The clinics that expressed interest worked with the research team, the CECP and patients, to develop their electronic PROM (ePROM) implementation plan. Two complementary implementation science frameworks were selected to guide the process and to identify the determinants of implementation of ePROMs at the level of patients, clinicians, and the organization. The objective of this study was to present the knowledge translation (KT) [14] and implementation science approaches that we used to inform the implementation of ePROMs across an integrated chronic pain network that includes primary, rehabilitation, and hospital-based care. Specifically, we present how we applied two implementation science frameworks to evaluate determinants of implementation, to guide the selection of strategies to implement ePROMs in clinical practice, and to select implementation outcomes.

## Application of theoretical frameworks for implementation in the context of the CECP network

To address the gaps in chronic pain management, the CECP developed a strategic plan to provide integrated care for individuals that included early referral to the right service based on individuals’ needs. In line with evidence-based practices, interdisciplinary teams were integrated in primary care clinics in four health regions, with referral to secondary and third lines of care for more complex chronic pain cases.

In order to successfully implement ePROMs to screen for patient service needs, develop individualized intervention plans, and monitor changes, results from two implementation research studies were integrated, one in primary care [15], and a one in tertiary and rehabilitation care [2, 11]. A steering committee reviewed the results of each study during four 1.5-h working meetings and developed and iteratively reviewed the implementation plan. Committee members consisted of a decision maker and clinician from each site, the director of the CECP, two research team members, and two patient partners met during the studies and at the end.

**Fig. 1:**
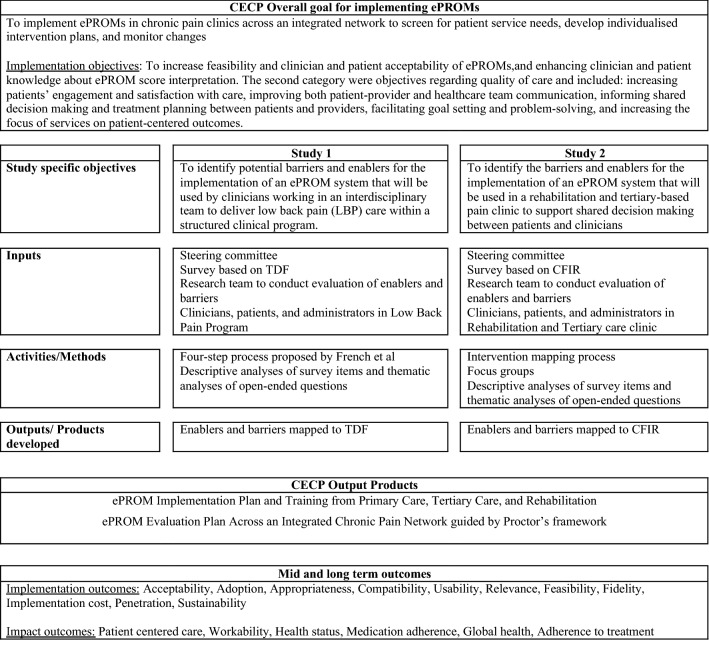
Logic model for the development of an implementation and evaluation plan of an ePROM system across the Center of Expertise in Chronic Pain integrated network

The choice of the theoretical implementation framework used in each study or setting was mainly driven by the specific clinical context, and the understanding of our team at the time about implementation research and its application to ePROMs (Fig. 1). In primary care, the Theoretical Domains Framework (TDF) was selected as it allowed for identifying determinants of implementation of ePROMs more at an individual clinician level and for developing theory-based behavioural change strategies to address barriers and train clinicians in the use and application of PROMs for patient care. In these settings, the main focus was to train individual team members to interpret and apply ePROM scores to treatment planning within a well-defined clinical process endorsed and supported by the healthcare organization. In tertiary and rehabilitation care, where there is more variability in care delivery processes, we needed to consider determinants related more to organizational change in addition to determinants of clinician and patient behaviour change. In these settings we used the Consolidated Framework of Implementation Research (CFIR) to evaluate clinician, individuals with chronic pain, and organizational factors that influence implementation of ePROMs.

In this paper, we present an overview of the use of each framework (Table 1) and how the use of these frameworks resulted in a final PROM implementation plan. The steering committee integrated results across both studies to inform PROM implementation and evaluation. The Proctor framework was used to guide the development of an evaluation plan for the implementation of PROMs. The details of the methodology and results for each study are presented elsewhere [2, 15, 16]. Here, we focus on the use of the frameworks and the resulting implementation plan to feedback PROM scores to patients and clinicians. Finally, we contrast the use of the TDF and CFIR frameworks, assess whether these frameworks resulted in identifying similar barriers, and present the final PROM implementation plan, in addition to the evaluation plan that will be used across all CECP settings.

**Table 1:** Summary of studies that evaluated ePROM determinants of implementation across an integrated chronic pain network

Study	Objective	Setting	Phase of ePROM intervention	Methods for KT intervention development	Data collection	Unit of analysis
Eilayyan et al. theoretical domains framework	To identify potential barriers and enablers for the implementation of an ePROM system that will be used by clinicians working in an interdisciplinary team to deliver low back pain (LBP) care within a structured clinical program	Primary care	Design, Implementation plan development, evaluation plan	French 5 steps	Survey based on the TDF with close and open-ended items	Clinician
Simeone et al. consolidated framework for implementation research	To identify the barriers and enablers for the implementation of an ePROM system that will be used in a rehabilitation and tertiary-based pain clinic to support shared decision making between patients and clinicians	Rehabilitation tertiary care	Design, Implementation plan development, evaluation plan	Intervention mapping	Survey based on CFIR with closed and open-ended items Focus groups with healthcare professionals and 2 patient partners	Patient clinician organization

## Application of the TDF in a primary care interdisciplinary program

### Objectives: primary care study

The objective of this study was to identify potential barriers and enablers for the implementation of an ePROM system that will be used by clinicians working in an interdisciplinary team to deliver low back pain (LBP) care within a structured clinical program.

### Study design and procedure: primary care study

To address the study objectives a four-step process proposed by French et al. (2012) [17] was used. The French approach starts with posing the question “who needs to do what differently?” and focuses on the actors implementing the intervention (ePROMs). Step 2 involves identifying the barriers and enablers to implementing the intervention. We chose the TDF framework for Step 2 as it was developed to mostly identify barriers to individual clinical behaviour change that matched the context of the chronic pain interdisciplinary teams. We also felt that the domains could be more readily mapped to behaviour change strategies (and mode(s) of delivery) to include in the ePROM implementation plan that could overcome the modifiable barriers and enhance the enablers (Step 3 of French et al.). Finally, Step 4 answers the question “How can behaviour change be measured and understood?”. We address this in the evaluation plan guided by the Proctor framework.

The TDF was developed and used to identify the barriers and enablers to clinical behavioural change among clinicians to inform the design of a theory-based ePROM implementation [18–22]. It includes 14 validated domains which are deemed to be the main contributors to behaviour change among clinicians: Knowledge, Skills, Social/Professional Role and Identity, Beliefs about Capabilities, Optimism, Beliefs about Consequences, Reinforcement, Intentions, Goals, Memory/Attention and Decision Processes, Environmental Context and Resources, Social Influences, Emotion, and Behavioural Regulation [23].

The 14 TDF domains were evaluated using an online self-administered survey that aimed to identify clinicians’ perceived barriers and enablers to using ePROMs. The survey included 30 questions adapted from validated TDF instruments [24, 25]. Items were rated on a 5-point Likert scale (strongly agree to strongly disagree). The survey also had 16 open-ended questions to obtain more information on clinicians’ opinions about the domains. A thematic analysis was conducted for open-ended responses to identify additional enablers and barriers.

### Results: primary care study

Eighteen of 25 invited health professionals from primary care interdisciplinary teams responded to the survey (response rate of 72%). Respondents included two physicians, six physiotherapists (PTs), three nurses, three psychologists, two occupational therapists (OTs), and two kinesiologists. The average age of the participants was 39 years (SD ± 7.7); 39% (7/18) were females, and the average number of years practicing their respective professions was 14 years (SD ± 8.4). Determinants identified as likely to restrict the use of PROM scores included skills, social/professional role and identity, goals, decision processes, beliefs about consequences, environmental context and resources, behavioural regulation, and social influence. The barriers and enablers identified for each domain are summarized in Table 2.

**Table 2:** Determinants to implementation of ePROMs in primary care using the TDF*

Domain	Barrier	Enabler
Knowledge		Awareness of the objectives of using ePROMs in clinic
Skills	New skills are required to successfully use PROMs in the management of patients with LBP	Having the skills needed to interpret the results of ePROMs
Social/Professional role and identity	Lack of clarification of clinicians’ professional role in relation to using ePROMs	Believing that using ePROMs is one of the clinicians’ role in clinic for individual patient management of LBP
Optimism		Expecting improved patient outcomes as a result of using ePROMs in the management of patients with LBP Optimism regarding the benefits of using ePROMs in the management of patients with LBP
Beliefs about consequences	Using ePROMs in clinical practice is not necessary to improve patient outcomes	Clinicians believe in the benefits of using ePROMs in the management of patients with LBP
Reinforcement		Having better patient health outcomes makes clinicians continue using ePROMs in the management of patients with LBP
Intentions		Clinicians’ commitment and intention to use ePROMs in the treatment of patients with LBP in the next three months
Goals	The plan of how to use ePROMs in clinical practice is not clear	The use of ePROMs in the treatment of patients with LBP is not more important and prioritized compared to only using clinical outcomes
Decision processes	The use ePROM scores is difficult in making treatment decisions	
Environmental Context and Resources		Lack of time to use ePROM scores in the clinical setting

*TDF Theoretical Domains Framework, *LBP* Low Back Pain

Enablers reported in the open-ended question included clinicians’ motivation to use PROMs, having access to patient scores with the interpretation, and having patients fill out the questionnaires electronically. Factors that clinicians identified that may reduce the use of PROMs included lack of knowledge, limited time, and limited access to patients ePROM results if some patients choose not to complete measures or if there are a lack of resources to compile patient data. Disadvantages for using ePROMs listed by clinicians included patients’ having difficulty understanding some PROM items, clinicians having a limited understanding of what patients’ scores mean, limited usefulness of PROM scores in reflecting patients’ needs, and having to manage large volumes of ePROM data.

## Application of the CFIR in rehabilitation and tertiary care

### Objectives: rehabilitation and tertiary-based care study

The objective was to identify the barriers and enablers for the implementation of an ePROM system that will be used in a rehabilitation and tertiary-based pain clinic to support shared decision making between patients and clinicians.

### Study design and procedure: rehabilitation and tertiary-based care study

This project employed an intervention-mapping (IM) process [26]to inform the development of a tailored implementation plan of an ePROM system at a hospital-based pain clinic. IM consists of six steps: 1. Needs assessment; 2. Definition of program objectives; 3. Selection of theory-based intervention methods; 4. Production and pretesting; 5. Adoption, implementation, and sustainability planning; 6. Process and effect evaluation. We chose the Consolidated Framework for Implementation Research (CFIR) as a theoretical framework to guide the IM steps. CFIR serves as a practical guide for systematically assessing potential determinants in preparation for implementing an innovation [27]. The details of this study are presented elsewhere [2, 15, 16]. In this paper, we focus on the methods and results related to application of the CFIR to identify the determinants, barriers, and enablers to implementing ePROMs.

A survey with open-ended questions about enablers/barriers to the implementation of ePROMs was administered to clinicians and patients. Our clinical partner on the project, a nurse pain specialist working at the hospital-based pain clinic, recruited a convenient sample of both patients and clinicians to complete a survey adapted to both groups of participants. Patients were asked to come one hour before or immediately after their appointment with their clinician to complete the survey and answer several open-ended questions. Clinicians actively caring for patients at the pain clinic were sent an email from our clinical partner on behalf of the research team inviting them to complete an online survey. To increase participation, three email reminders were sent one week apart.

Based on the survey results, a focus group guide was developed, and focus groups were conducted with clinicians to explore their clinical practices related to the use of PROMs, and to deepen our understanding of the main perceived barriers and enablers to their use in the management of CP. Focus groups were digitally recorded and transcribed.

Quantitative survey data from clinicians and patients were analysed using descriptive statistics. The qualitative data included the open-ended questions of the patient survey and the content from the focus groups with clinicians and were analysed using deductive content analysis [28] based on core CFIR constructs. Two researchers independently conducted the analysis. All emerging themes were coded, and the two investigators continuously compared their analytical interpretations to identify the similarities and differences in the participants’ experiences to arrive at a common understanding. The results of the qualitative and quantitative analyses were reviewed with the study steering committee and three additional clinicians from the pain clinic. Together, they defined the program objectives, and synthesized the barriers and enablers identified from the survey and focus groups to be considered in the design and implementation plan of the ePROMs system.

### Results: rehabilitation and tertiary-based care study

The survey was completed by 13 clinicians. Four were males and nine females. Five of the clinicians were psychologists, three nurses, two anaesthesiologists, two medical doctors, and one physiotherapist. A convenient sample of twenty-four patients, 12 males and 12 females responded to the survey with an average experience with CP of 9.8 ± 8.5 years. Overall, four focus groups were conducted, and a total of 10 clinicians were interviewed. Clinicians and patients were representative on average of those working or receiving care in the clinic.

Determinants were classified as barriers or enablers across the CFIR domains as shown in Table 3. Several enablers related to Intervention Characteristics that may facilitate implementation included designing the ePROM system in a manner that is easy for clinicians to use and meets patient needs (e.g. font, brightness), providing users with evidence about the clinical validity of PROMs, selecting PROMs that are easy to complete and interpret, and enabling access to ePROM systems using all forms of devices. Barriers related to the intervention included concerns with data privacy (e.g. who would be allowed to view PROM scores) and cost of implementing PROMs.

**Table 3:** Determinants to implementation of ePROMs in rehabilitation and tertiary care using the Consolidated Framework for Implementation Research

CFIR Domain	Barrier	Enabler
Intervention characteristics		
Evidence strength & quality		ePROMs will provide valid clinical information
Complexity	Filling ePROMs can be too much effort and time for some patients	ePROM system easy to use for both patients and clinicians Include in ePROM, ePROMs simple to complete, short and easy to interpret
	Privacy/ confidentiality concerns	
		ePROMs easy to understand by patients: goes in complexity
Design quality and packaging		Provide clinicians with rapid access to ePROM scores
		Align ePROM design to patients’ needs (font size, brightness, etc.)
		Design ePROM system that can be accessed from various devices (smart phone, tablet, computer)
Cost	Additional funding needed to support implementation of ePROMs (equipment, administrative)	
Outer setting		
Patient needs and resources	Not all patients have access to IT Low level of literacy of patients	Patients interested in completing ePROMs
	Insufficient patient cognitive abilities to be able to complete ePROMs	
	Patients not understanding English or French	
Inner setting		
Implementation climate	Lack of time to look at ePROMs results at Each patient’s visit	Beliefs of senior management of the importance of ePROM Implementation
Readiness for implementation		Onsite IT support
		Having human resource support to help patients fill out ePROMs
		Training [clinicians] on the use of the ePROM system and interpretation of ePROM results
Characteristics of individuals		
Knowledge and beliefs about the intervention	Clinicians’ little familiarity with ePROMs Usefulness and relevance of an ePROM system	Familiarity with technology
	Information provided by ePROMs will not be comprehensive	
	ePROMs do not always meet the information needs of patients	
	Using ePROMs may decrease patient satisfaction with healthcare services	ePROMs will help with pain management and improve [patient] engagement
	Patients not willing to provide accurate information	
Individual stage of change	Reluctance of some clinicians to use the ePROM system: individual stage of change	Motivation and interest of some clinicians for an ePROM
Motivation		
Process		
Planning		Completion of ePROMs aligned with clinical process: while patient in waiting room
		“Top down” implementation Involvement of multidisciplinary team in development of an implementation plan (data collection and clinical process to use ePROM scores) Project manager for data collection process and clinical use of ePROMs information
Engaging		Interest and support of stakeholders in ePROM implementation
		ePROMs will be filled by patients if clinicians motivate patients to do so

In the outer setting, barriers were related to patient dimensions including PROMs that matched individuals’ literacy level, the language spoken if it was not English or French, and having access to technology to be able to complete ePROMs at home or in the clinic so that scores would be readily available during the clinical encounter. There were also concerns that patients with limited cognitive abilities may not be able to understand PROM questions and responses.

Inner setting-related enablers included senior management emphasizing the importance of having onsite IT support, having human resource support to help patients complete ePROMs, providing clinicians with rapid access to results, and training on the use of ePROMs including a guide to interpret the results, and information on the validity of the measures used in the ePROM system. On the other hand, the perceived increase in workload and time associated with implementing ePROMs were considered as barriers to the use of an ePROM system.

Clinicians’ knowledge and beliefs that can facilitate implementation included perceptions that were important to use ePROMs and that sharing ePROM scores with patients will help with improving patient engagement and in turn pain management, and clinicians’ familiarity with the ePROM intervention. The counterpart barriers to these enablers included clinicians’ limited knowledge of the benefits of PROMs and how to apply them in clinical care, limited perceived usefulness of an ePROM system, and clinicians’ perception that PROMs may not be comprehensive and meet the information needs of patients. Clinicians also expressed concerns that use of PROMs may decrease patient satisfaction with health services, and that some patients may not provide accurate information on PROMs.

Finally, several determinants that were identified as likely facilitating the process of implementing PROMs included garnering support for ePROMs from all stakeholders, deciding at the department [leadership] level to implement ePROMS (top down), involving the multidisciplinary team in the development of the ePROM system, engaging clinicians in motivating patients to complete ePROMs, aligning implementation of ePROMs to the clinical workflow, and having a project manager in the clinic to support data collection and use of ePROM information in clinical care.

## ePROM implementation plan and training from primary care, tertiary care, and rehabilitation

The steering committee reviewed all information regarding the determinants of implementation of ePROMs identified across both studies and reviewed the objectives of the ePROM system implementation plan across the integrated pain network. The program objectives encompassed two broad categories. The first was adoption of PROMs, mainly, increasing feasibility and clinician and patient acceptability of ePROMs, and enhancing clinician and patient knowledge about ePROM score interpretation. The second category were objectives regarding quality of care and included increasing patients’ engagement and satisfaction with care, improving both patient-provider and healthcare team communication, informing shared decision making and treatment planning between patients and providers, facilitating goal setting and problem-solving, and increasing the focus of services on patient-centred outcomes.

The steering committee agreed to pursue the following steps for implementation at an organizational level. First, develop a business plan to incorporate the costs related to the ePROM system by directly integrating resources needed into the sites’ budget. This will be accomplished by examining potential cost savings associated with using ePROM scores to triage patients more efficiently and administrative time needed to complete paper-based forms for standardized and non-standardized patient information. The second organizational change recommended was to link ePROM scores to the clinic’s electronic medical record to facilitate integration of ePROM scores into the clinical workflow.

The steering committee defined the following process for facilitating implementation at the level of the clinical unit: administrative staff will send an email to patients containing the link to the ePROM questionnaire 2–4 weeks prior to the first visit or follow-up appointment. The patient will be invited to complete ePROMs at home 1 week prior to first visit or follow-up appointment. If PROMs were not completed at home patients will be encouraged by email and/or telephone to arrive before his/her appointment to complete ePROMS at the clinic.

The ePROM results will be shared with clinicians and patients through an online feedback report before the clinic visit. The feedback report includes patients’ scores over time for each measured domain, and the interpretation of each PROM score using graphs and text to indicate the current scores and the changes over time in each domain. Prior to the first visit or follow-up appointment, clinicians will be prompted to review the results in preparation to reviewing the scores with patients during the clinical encounter.

Evaluation of the determinants played a critical role in deciding on the implementation strategies that will be included in the development of a KT intervention that will increase the use of ePROMs. The process of deciding on which strategies to include in the KT intervention are presented elsewhere [2, 15, 16]. The main selected strategies were an interactive training program. The program will include educational/ instructional material on the selection, application, and interpretation of PROM scores and the clinicians’ and patients’ roles in using PROMs to plan treatment and monitor changes in outcomes in collaboration with patients. The program will also include a half-day training workshop on the use of PROMs in clinical practice that will include interpretation of feedback reports of individuals’ PROM scores.; The second strategy will be designating an opinion leader to provide clinicians with one-on-one coaching when needed to support the implementation of the KT intervention components and to provide coaching to clinicians on the use of PROMs. Table 4 presents the final interactive training program that will be adopted by the McGill RUISSS CECP.

**Table 4:** Description of training components for implementation of ePROMs

Training components	Description
Educational material	For clinicians, material will target: Information on selection of ePROMs (to supplement core set chosen by clinical sites) Interpreting ePROM score reports Interpreting the minimal clinically important change Linking ePROM scores to treatment components (each team is currently working on guidelines to give global guidance to team members) that should be implemented Educational material will also be presented in a Webinar and Video that will be used as booster sessions after the workshop Encourage clinicians to share with their patients the value of ePROMs to assist with collaboratively planning their care
Workshop	Interactive workshops in small groups led by a local clinical champion and facilitated by a research team member Goal is to: Reinforce the clinician’s role about the use of ePROMs within their clinical practice Identify practice needs of clinicians and identify links with how ePROM scores can provide potential solutions For clinicians to develop the knowledge and skill needed to use ePROMs in clinical care and practice applying information in the educational material and feedback reports To practice communicating and discussing ePROM scores with patients
Feedback reports of individual patient scores and of clinician use of the ePROM system	Developing and using patient ePROM score reports that include: The patients’ scores and trends over time Interpretation of scores Treatment algorithm (first proposed action plan) Develop reports that summarize clinicians’ use of the ePROM system, viewing and discussing results with patients, and treatment decisions made based on ePROM scores
Opinion leader	Identifying and training an opinion leader in each clinic to: Reinforce the clinicians’ role and potential benefits of using ePROMs in clinical practice Facilitate the use of ePROMs (one to one coaching) Review use of ePROM scores with health professionals for specific case, and across the clinicians’ full case load

## ePROM evaluation plan across an integrated chronic pain network

Guided by the Proctor framework of evaluation, [13] the steering committee agreed on a set of outcomes that will be used to evaluate implementation and impact of using PROMs. The PROMs that will be implemented as part of patient care are those that are part of a CORE set prioritised by patients and clinicians [29] using PROMIS measures [30]. The framework “positions implementation outcomes as preceding both service outcomes and client outcomes, with the latter sets of outcomes being impacted by the implementation outcomes” [13]. The selection of outcomes was guided by the CECP objectives for using ePROMs, and the interrelationship of outcomes we expected to be impacted by adoption of the ePROM intervention. For example, we would expect to see the strongest impact on patient outcomes (e.g. general health, pain interference, work ability) as the ePROM uptake and sharing and discussion of scores with patients increases in a clinical setting. The Proctor framework derives service outcomes from the six quality improvement aims set out in the reports on crossing the quality chasm: the extent to which services are safe, effective, patient-centred, timely, efficient, and equitable [31, 32]. Table 5 presents the implementation and impact outcomes that will be used to evaluate the ePROM intervention across the McGill RUISSS CECP integrated network.

**Table 5:** Evaluation Plan for the ePROM intervention across an integrated chronic pain network

Outcome	Definition	Measure/Indicator
Implementation outcomes		
Acceptability	Satisfaction with the ePROM system	Acceptability of Intervention Measure [1, 2]
Adoption	Utilization of the ePROM system	Adoption of Information Technology Innovation Measure % of clinicians using the ePROM system
Appropriateness	Perceived fit of the innovation: compatibility, usability, relevance	Intervention Appropriateness Measure [1, 2]
Compatibility	The degree of tangible fit between meaning and values attached to the intervention by involved individuals, how those align with individuals’ own norms, values, and perceived risks and needs, and how the intervention fits with existing workflows and systems [27].	Survey of Organizational Readiness for E-Health Open-ended questions
Usability	How useful, usable, and satisfying a system is for the intended users to accomplish goals in the work domain by performing certain sequences of tasks [33]	End-User Computing Satisfaction Questionnaire [3] Open-ended questions
Relevance	The cognitive impact, as well as any use and patient health benefit associated with the information derived from PROM scores	Information Assessment Method [4]
Feasibility	Actual fit of the innovation	Feasibility of Intervention Measure 2] System's web analytics, administrative data Examples of indicators: % of patients to whom an ePROM link was sent % of patients that filled ePROM before appointment
Fidelity	Innovation delivered as intended	Administrative data: % of patients admitted to the program who completed ePROM as planned
Implementation cost	Marginal cost, cost-effectiveness, cost–benefit	Cost of developing and implementing the ePROM system Screening for a program allocation Number of visits
Penetration	Reach, spread, access of the service	Administrative data Number of actual users divided by the total number of persons eligible to use the system
Sustainability	Maintenance, continuation, durability, incorporation, integration, institutionalization, sustained use	Sustainability questionnaire
Impact outcomes		
Patient-centred care	Providing care that is respectful of, and responsive to, individual patient preferences, needs and values, and ensuring that patient values guide all clinical decisions	Patient Assessment of Chronic Illness Care + [5, 6]
Workability	Work ability and productivity	Are you working at a physically less demanding job now because of your pain? What is your current work status? How long after you received treatment for pain did you return to work?
Health status	Individuals’ general health perceptions	EQ-5D-5L [7] VAS [8]
Medication adherence	Adherence to prescribed medication	Morisky 8 Medication Adherence Questionnaire [9, 10]
Global health	Individual’s physical, mental, and social health	Global health: PROMIS short form [11]
Adherence to treatment	Adherence to clinicians’ recommendations	Single item VAS [8]

## Discussion

In this review of two studies, we demonstrated the use of the TDF and CFIR as guiding frameworks to evaluate determinants of implementation of ePROMs. As recommended by Birken et al. (2017), we explicitly specified the rationale for using the TDF or the CFIR to contribute to our understanding of each framework, its scope, and its limitations of using both frameworks to implement PROMs. Using theoretical frameworks facilitates identifying barriers and enablers to implement ePROMs in clinical settings and guides the development of theory-based interventions aimed at closing the gaps between knowledge and practice. The results of this study provide researchers and clinicians that are planning to monitor implementation of ePROMs an understanding of how the TDF and CFIR can be applied and highlight possible barriers and enablers that will likely need to be addressed.

The contrast and integration of results from both studies provided us with rich insight into the application of both approaches to the implementation of PROMs in clinical care. Similar to previous studies, our rationale for selecting the TDF or CFIR was driven by the purpose at the time for evaluating the determinants of implementation. The TDF was used in the context of an interdisciplinary team perspective, where each team member had to consider use of PROM scores for creating an intervention plan related to their discipline, and to link the resulting treatment plan to the overall program delivered by all team members in collaboration with the patient. The TDF targeted well the individual-level determinants of implementation that needed to be considered. Also, the clinical process of the LBP program was well defined and endorsed by the healthcare organizations where these programs were implemented as it was part of the provincial CECP Action Plan. Using ePROMs in these programs was endorsed and was an organizational expectation from the start. In contrast, we used the CFIR in rehabilitation and tertiary care where organizational-level factors were felt to play an important role in the adoption of ePROMs. The CFIR operationalized the individual characteristics of clinicians and patients (e.g. knowledge & beliefs), and the organizational and process factors that are critical in planning the implementation of PROMs.

Interestingly, both frameworks provided similar results with respect to healthcare providers’ knowledge and behaviour. These included the importance of considering clinician’s attitudes and perceptions of the benefits of using ePROM scores, such as believing they will have a positive impact on patients’ outcomes. The CFIR captured more barriers in relation to knowledge and beliefs including clinicians’ beliefs that PROM scores may not be comprehensive [of patients needs] and the perception that using PROMs may decrease patients’ satisfaction with care. Intention and motivation to use PROMs were identified in the TDF and CFIR study, respectively. These individual clinician characteristics identified in our studies are similar to those reported in previous work [34–38]. Both frameworks were also consistent in identifying clinicians’ knowledge and experience in using PROMs, and difficulties with interpreting PROM scores as barriers, similar to previous work [35, 39, 40].

Where the TDF and CFIR frameworks differed was in identifying organizational-level determinants, which the CFIR was able to capture because of the explicit questions asked linked to the CFIR domains. This was reported in previous studies that contrasted the use of both frameworks for implementing healthcare interventions [41–43]. Some consensus among coders in each of our studies required discussion, as some statements from the qualitative data were not easy to classify in either framework. However, this was more frequent for the TDF. For example, we felt that statements related to the relationship between clinician and patient were harder to map to the TDF framework. In the CFIR we classified “PROMs will be filled by patients if clinicians motivate patients to do so” as best fitting under ‘process’ in the CFIR, and there is no equivalent domain in the TDF.

In all settings we started with evaluating the current use of measures, what improvements in measurement were required, and asking clinicians and patients about their needs in terms of patient care to link use of PROMs to these needs [11, 44]. This was a key first step to understand the baseline practices of the sites and to tailor the knowledge translation interventions for ePROM implementation and training content to the readiness of the sites to begin using ePROMs. We also had knowledge exchange sessions with clinicians regarding the measures they were currently using and whether there were more efficient ePROMs with more comprehensive coverage of domains, stronger psychometric properties, and available evidence for interpretation of ePROM scores.

The resulting KT and training intervention elements will form a multi-component intervention, which can address multiple gaps and barriers [45], increase clinicians’ knowledge and skills, and foster best practices. A previous study [11] identified the most important PROMs as perceived by clinicians and patients that will form the core set of PROMs that will be included across all sites. As clinical programs learn from their experience with this core set, the use of ePROMs will be continually improved to include additional measures, linkages between ePROMs and electronic medical records, and optimization of the timing of administering ePROMs. The ongoing evaluation will be essential for informing ongoing improvements and sustainability of implementation and use of ePROMs.

Lastly, the importance of ePROMs feedback reports was emphasized across all our studies. All clinicians, to save time and facilitate the sharing of ePROM results among clinicians and patients, highlighted the format and integration of these reports in the electronic medical record. Feeding back the scores to clinicians and patients may increase their belief in value of using PROMs in clinical practice, improve the communication between patients and clinicians, and empower individuals with chronic pain to actively participate in managing their health condition, which may lead to better treatment adherence [46, 47]. We will qualitatively evaluate the determinants of ePROM implementation over time to iteratively improve ePROM implementation elements including feedback reports, KT and training, and integration of ePROMs across the integrated chronic pain network to facilitate uptake by each care setting that is added as we scale up the use of ePROMs to support patient-centred care.
